# Progression of Scoliosis after Skeletal Maturity in Patients with Cerebral Palsy: A Systematic Review

**DOI:** 10.3390/jcm13154402

**Published:** 2024-07-27

**Authors:** Klaas Victor, Pierre Moens

**Affiliations:** Department of Orthopaedics, University Hospitals Leuven, 3000 Leuven, Belgium; pierre.moens@uzleuven.be

**Keywords:** scoliosis, cerebral palsy, curve progression, skeletal maturity

## Abstract

**Background:** The progression of scoliosis has been observed in skeletally mature patients with cerebral palsy (CP). The aims of this systematic review were to determine the incidence of curve progression of untreated scoliosis after skeletal maturity, to estimate the average annual increase and to identify factors that influence the progression. **Methods:** A systematic literature search was performed in PubMed, Embase and the Cochrane Library for original research articles published between 1968 and May 2024 with a retrospective, prospective or cross-sectional design, investigating CP patients that were followed up beyond the age of 15 years. The search was limited to articles in English, French, German and Dutch. Articles were excluded if the study population concerned neuromuscular diseases other than CP. After an assessment of the methodological quality of each study, estimates of annual curve progression and the effect of the investigated risk factors for progression were recorded systematically and synthetized. **Results:** Fifteen studies met the inclusion criteria, resulting in a total sample size of 2569 participants. The study populations of the included original research articles were small and heterogeneous in terms of patient age and the type and severity of CP. Curve progression after skeletal maturity occurred in all included studies. A greater curve magnitude at the end of adolescence and a severe motor deficit (an inability to walk or GMFCS IV-V) were identified as significant risk factors for the progression of scoliosis after skeletal maturity. If at least one of these risk factors was present, scoliotic curves progressed after skeletal maturity in up to 74% of patients, with an average annual increase of 1.4 to 3.5 degrees per year. No significant association was found between curve progression and the physiologic type of CP, the type of scoliotic curve, previous hip surgery, positioning and gravity, weight and length, sex, epilepsy, or pelvic obliquity. Findings on the effect of hip instability were inconsistent: a positive correlation was found with the progression of scoliosis overall, but not after skeletal maturity in particular. A significant selection bias should be considered in the calculation of average annual curve progression, as patients that received interventions to halt curve progression were excluded from follow-up. **Conclusions:** The identification of risk factors in patients with CP and scoliosis can aid in predicting curve progression and managing follow-ups in clinical practice. Based on the findings in this review a radiographic follow-up once every 3 years is recommended for skeletally mature CP patients with at least one risk factor, and once every 5 years if no risk factors are present.

## 1. Introduction

Cerebral palsy (CP) is defined as a group of permanent movement and postural disorders, caused by a non-progressive disturbance in the pre-, peri- or postnatal development of the brain [1]. Tone abnormalities are the primary symptom and result in a range of impairments, from gait and sitting disturbance to a complete inability to walk or sit [2]. Scoliosis, defined as a Cobb angle exceeding 10°, is highly prevalent in patients with CP, with an overall prevalence of 18–29% [3,4], ranging up to 64% in groups with severe motor impairment or a higher age [4,5]. Scoliosis is a major contributing factor to gait disability, sitting imbalance and impaired upper limb function in these patients [6]. Furthermore, it is a common cause of pain [2] and pulmonary restrictions [7]. CP is an incurable disease, but treatment strategies focusing on the management of scoliosis can take away chronic discomfort, increase functioning, facilitate participation and improve quality of life in patients with this condition [2].

The progression of scoliosis occurs most frequently during growth but has also been observed after skeletal maturity in patients with CP [8]. Despite the importance of identifying individuals at risk for curve progression in order to guide strategies for both follow-ups and the timing of interventions, very few studies have tried to elucidate the natural history of scoliosis after growth maturity in patients with CP [9]. To date, physicians have been unable to predict curve progression in skeletally mature CP patients. The aims of this systematic review are as follows: (1) to determine the occurrence of the progression of scoliosis after skeletal maturity in CP patients; (2) to estimate the annual changes in scoliotic curve if progression occurs; and (3) to identify the factors that influence the progression of scoliosis in skeletally mature CP patients.

## 2. Materials and Methods

The PRISMA statement for reporting systematic reviews [10] was used to set up a search and selection strategy, to perform data extraction and to address the interpretation of the results. A systematic literature search was performed in PubMed, Embase and the Cochrane Library from 1968 to May 2024. The search algorithm as shown in Table 1 was designed to yield a maximum number of articles in the PubMed database and then modified to search the other databases.

The inclusion criteria were as follows: (1) the article is an original retrospective, prospective or cross-sectional study, (2) the study population fully or partially consists of patients with CP and untreated scoliosis, (3) one of the outcome measures is the magnitude of the scoliotic curve, and (4) at least a part of the study population is older than or followed up beyond the age of 15 years. Articles were excluded if the study population only concerned neuromuscular diseases other than CP. The search was limited to articles in English, French, German and Dutch.

This study was registered prior to commencement with SCONE, the ethical platform of KU Leuven Group Biomedical Sciences (registration number MP002986).

**Figure 1:**
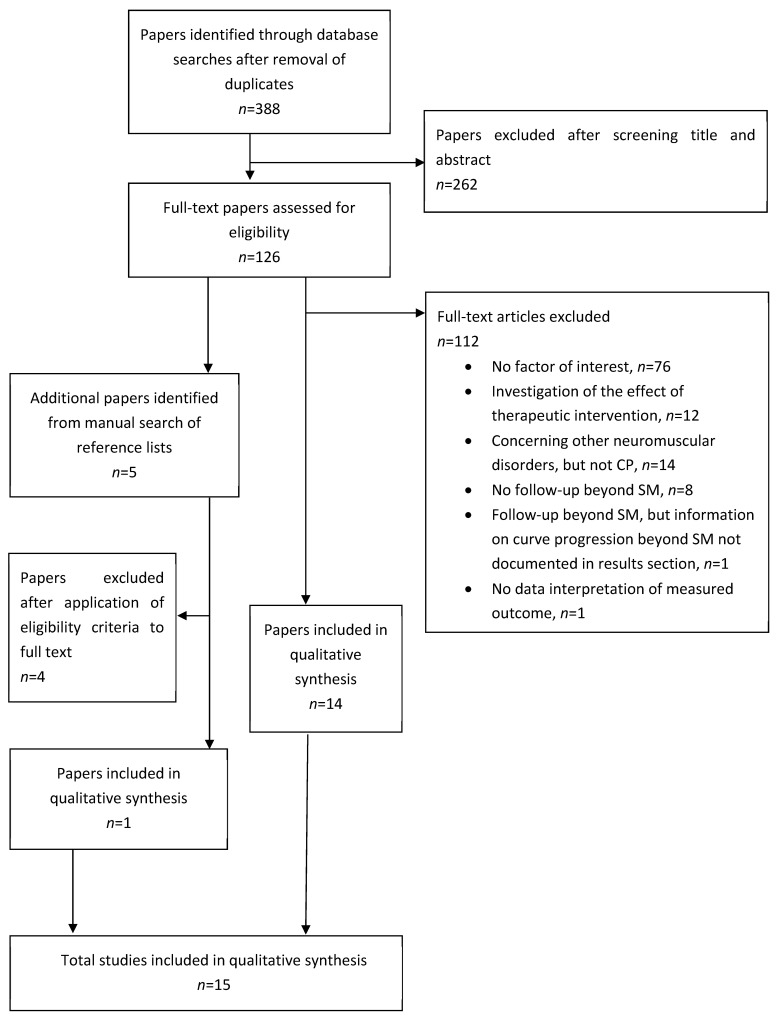
Flowchart of the review process. SM = skeletal maturity.

After the execution of the search algorithm in the three mentioned electronic databases and after the removal of duplicates, a total of 388 unique original research articles was obtained. Five additional articles were identified by the screening of reference lists. One original observational study was excluded because it reported on the development of a deformity documentation approach [11], without data interpretation to quantify curve progression. One study provided a follow-up beyond skeletal maturity, but information allowing one to draw conclusions on curve progression after skeletal maturity was lacking in the results section [12]. Other causes of exclusion are reported in Figure 1. Out of the 393 articles identified by the systematic search, 15 were found eligible for inclusion. Two authors were involved in the review process. Articles were only included if approved by both reviewers. No automation tools were used in the screening process.

A quality assessment of the included studies was performed in accordance with the 2011 Oxford Centre for Evidence-Based Medicine Levels of Evidence [13]. All the included articles were level III or IV studies. To obtain a more precise estimate of the evidence for each of the included studies, an assessment of their methodological quality was set up. Checklists from the National Institute for Clinical Excellence (NICE) [14], Joanna Briggs Institute (JBI) [15,16] and the Institute of Health Economics (IHE) [17] were evaluated for application to the included studies, as recommended by the Journal of Evidence-Based Medicine [14]. The Quality Assessment Tool for Case Series (QAT-CS) of the IHE [17] appeared to be the most applicable to the 15 studies. It was developed with a Delphi approach, suitable for case series, more elaborate than the NICE checklists and is one of the most recently developed tools. The assessment criteria were optimized to make them relevant and applicable to the included research papers on the progression of scoliosis in a CP population. A dichotomic or trichotomic score was attributed to each of the criteria (Table 2), allowing a maximum total score of 26 points. Each of the 15 studies was subsequently scored according to the modified QAT-CS criteria (Table 3) by a single reviewer. The risk of bias was similarly assessed through the QAT-CS criteria.

Data from each of the 15 included articles were extracted by a single reviewer using a standardized template, recording the study design, level of evidence, sample size, patient age, type and severity of CP and information on curve progression and investigated risk factors for progression. In case of conflicting information between different studies, a conclusion was drawn by lending increasing weight to studies with a higher methodological quality.

## 3. Results

Out of the 393 articles identified by the systematic search, 15 were included for quality assessment (Table 3) and standardized data extraction. The results on the incidence and rate of curve progression after skeletal maturity and its risk factors are summarized in Table 4.

### 3.1. Study Type and Level of Evidence

Fourteen studies were classified as level IV and one study as level III according to the 2011 Oxford CEBM Levels of Evidence [13]. Six studies used a prospective or cross-sectional study design [3,5,20,23,25,29]; the remaining nine studies were retrospective. The quality assessment showed a mean QAT-CS score of 16. The highest study in rank scored 19 points on the QAT-CS scale [18]; the lowest score was 8 points [29].

### 3.2. Characteristics of the Studied Populations

The studied groups in the included articles ranged from 34 to 962 participants. The average age of the participants and type and severity of CP differed between and within the various study populations. In six studies, all the patients were followed into adulthood after reaching skeletal maturity [8,19,20,21,24,28]. In the other studies, the population was mixed, also including groups of infants, children and adolescents without follow-up after having reached skeletal maturity. Multiple physiologic types of CP (spastic, dyskinetic, atactic) were represented in the study populations of most of the studies, whereas five articles investigated patients with the spastic type of CP only [8,18,22,26,28]. Five studies only included patients with severe CP (GMFCS ≥ level IV or quadriplegic patients only) [21,22,26,27,28]. Seven studies investigated an institutionalized population of CP patients [5,8,20,24,26,28,29].

### 3.3. Incidence and Rate of Progression of Scoliosis after Skeletal Maturity

Five studies described curve progression in study populations that solely consisted of patients followed into adulthood [8,19,20,21,24]. Saito et al. (QAT-CS 13) found that 73% of institutionalized patients with spastic CP and scoliosis have a continuing progression of their curve after the age of 22 years [8]. A similar incidence of curve progression (74%) was noted by Oda et al. (QAT-CS 17) in patients with severe CP [21]. In a more evenly distributed population including patients with both mild and severe motor impairment, Yoshida et al. (QAT-CS 18) found that 32.5% had more than 10° degrees of curve progression after the age of 20 years [19]. Thometz and Simon (QAT-CS 16) observed that the increase in Cobb angle after skeletal maturity was more than 1 degree per year in 78% of their study population, comprising patients with both mild and severe motor impairment. The maximum rate of progression was 3 degrees per year [24]. Oda et al. (QAT-CS 17) reported that 38% of patients with severe motor impairment (GMFCS IV and V) showed a curve progression of more than 1 degree per year [21]. Majd et al. (QAT-CS 17) measured an average increase of 2.5 to 3.5 degrees per year in a population that entirely consisted of skeletally mature CP patients (Risser grade V) [20]. The spastic, dyskinetic and mixed type of CP were all represented in this study, but all the participants were institutionalized patients.

Three studies reported on cohorts not solely consisting of patients that were followed into adulthood [3,18,29]. The average annual curve progression was 3.4° per year in a group of 42 CP patients with GMFCS level IV-V. This study had the highest level of methodological quality (QAT-CS 19), but included both skeletally mature patients and skeletally immature children [18]. Robson (QAT-CS 8) and Hägglund et al. (QAT-CS 14) found an increasing incidence of scoliosis with age, but did not specify skeletal maturity in their patient populations [3,29].

Only one study [28], with a low methodological quality (QAT-CS 11), did not find a positive correlation between increasing age and Cobb angle in skeletally mature patients. The study design was not longitudinal, and compared the average age in two groups that were discerned by the severity of the scoliotic curve. The groups were not balanced in number or patient characteristics.

### 3.4. Defining Significance of Risk Factors for Curve Progression

The average rate of curve progression differed significantly between studies and between the different groups of each study population. This was a consequence of small sample sizes and the widely differing characteristics of the studied groups. A systematic extraction of data concerning participant features in the included studies and their potential correlation with increased curve progression resulted in a synthesis of the current available evidence for factors associated with curve progression (Table 4). Depending on the coherence of the findings between the different studies, continuous and categorical variables with a potential effect on curve progression were classified into two groups.

A studied variable was classified as a “significant risk factor for progression” according to the following:

- The effect of this factor was investigated by at least 5 original research reports;
- All the reports or all but one report investigating the potentially influencing variable conclusively found an association of this variable with curve progression;
- At least two of these articles proved a statistical significance, of which one had a QAT-CS score equal or above the mean of 16.

Variables were classified as “no significant association found” according to the following:

- The findings of the articles investigating the variable were inconclusive;
- Only one article investigated this variable and did not find a significant association.

### 3.5. Significant Risk Factors for Progression

#### 3.5.1. Magnitude of the Curve at the End of Adolescence

Five of the included studies investigated whether the magnitude of the scoliotic curve in adolescence or at the completion of growth could predict future curve progression [8,19,21,24,26]. Three studies had a QAT-CS score above average and found a statistically significant relation between a greater scoliotic curve in adolescence and a faster rate of curve progression in adulthood.

Oda et al. [21] (QAT-CS 17) identified three patterns of progression rate depending on the curve magnitude at 15 and 18 years of age. Patients with a curve greater than 50° at the age of 15 showed an average progression rate (3.0 degrees per year) that was at least six times higher than in the group with a curve less than 50° at 15 years (0.1–0.5 degrees per year). In the group with curves < 50 degrees at 15 years, a second significant differentiation could be made at the age of 18 years. If the CA exceeded 20° at the beginning of adulthood, an average progression rate of 0.5 degrees per year was observed, whereas curves smaller than 20 degrees showed no continuing progression into adulthood. Despite the small sample size of 34 patients, the data were statistically significant. Only patients with a GMFCS classification of IV or V were included.

Thometz and Simon (QAT-CS 16) found a statistically significant difference in curve progression at the same benchmark of 50 degrees (CA) in patients with mild or severe motor impairment [24]. They observed a 1.75-fold increase in progression rate when comparing patients with CA < 50 degrees to patients with CA > 50 degrees at the attainment of skeletal maturity. The average annual increase was 0.8 degrees per year in the first group and 1.4 degrees per year in the second group. In this study, skeletal maturity was defined by Risser’s sign 5 and determined radiographically in each patient.

Two studies found a benchmark at 40° CA for differentiation of future curve progression. A publication in *The Lancet* 1998 (QAT-CS 13) showed that 85% of patients with spastic CP with a scoliotic curve of >40 degrees at 15 years would have a progression beyond 60 degrees in adulthood [8]. If the CA was less than 40 degrees by the age of 15, only 13% would develop a curve > 60 degrees in adulthood, and 54% would have a stagnation of their scoliotic curve through adulthood. However, the statistical significance of their data was not reported. Participants of this study had spastic CP and were followed up to a mean age of 25.1 years. Gu et al. (QAT-CS 15) observed a higher rate of progression in individuals with CA > 40° at the age of 12 years, whereas scoliosis was unlikely to progress if the CA did not exceed 40° at that age [26]. The results were statistically significant. Their study population consisted of patients up to 18 years of age, but they did not define curve progression in relation to skeletal maturity. All patients had severe CP (GMFCS IV-V).

Yoshida et al. found that a CA of >30 degrees by the age of 10 years was a significant risk factor for progression during growth. The CA at growth maturity was also 24 degrees larger in patients showing curve progression in adulthood than in patients without curve progression after the age of 20 years [19]; however, it was statistically non-significant.

#### 3.5.2. Initial Severity of Motor Impairment

Eight out of nine studies that investigated the influence of the severity of motor disability demonstrated that the scoliotic curve on radiographs progressed to a greater extent in the groups with a lower functional level. Two studies proved the statistical significance of their results [18,28].

Lee et al. [18] scored the highest of all the studies included in the quality assessment (QAT-CS 19) and observed an average progression of 3.4 degrees per year in patients with GMFCS level IV-V, whereas no significant progression was seen in patients with GMFCS I-III. Their study population consisted of 184 participants with spastic CP, and the results were proved statistically significant. The status of triradiate cartilage and Risser’s classification were determined for all study participants. The population was mixed, with 42 patients out of 184 classified as Risser 4 or 5.

Thometz and Simon and Saito et al. did not use the GMFCS classification to grade functional ability. Both articles found a higher risk of curve progression for patients without walking ability, in comparison to those who were able to walk [8,24]. Thometz and Simon (QAT-CS 16) measured a steady, almost linear curve progression after skeletal maturity (Risser 5) in the group of non-walking patients and a slower progression in the group of walkers. Both groups had a curve magnitude of >50° at skeletal maturity. A total of 37 patients with spastic CP were investigated by Saito et al. [8] Scoliosis progressed beyond 60° after skeletal maturity in all bedridden patients, in 29% of the patients able to sit and in none of the patients that were able to walk. Assigning study groups by degree of body involvement, 67% of the patients with total body involvement progressed beyond a CA of 60° in adulthood. Only 18% of patients without total body involvement showed progression to such an extent in adulthood. The study had a QAT-CS score of 13, and the limited sample size did not allow testing for the statistical significance of these results.

Two studies by Bertoncelli et al. (QAT-CS 17, 16), which accounted together for a total of 120 patients with different types of CP, also showed that participants who were able to walk independently or had a higher level of gross motor function (GMFM-88 > 30.1) were more likely to have non-progressive scoliotic curves that remained <40 degrees CA [23,25]. GMFM-88 is a tool for the clinical assessment of gross motor functioning in patients with CP. Activities, from lying and sitting up to walking, running and jumping skills, are scored. The total calculated score is a percentage. The higher this score, the better the functional abilities of the patient [30]. Patients that could not walk independently or had a lower functional level (GMFM-88 < 18.4) were at a higher risk of scoliosis progression beyond 40 degrees. No statistical significance was found for an independent association of walking inability with curve progression. It is important to note that these patients were not followed up beyond the age of 18 years and no information on skeletal maturity was provided. The mean duration of follow-up was 5.3 years.

Kalen et al. (QAT-CS 11) found that greater scoliotic curves in adulthood were associated with a significantly lower percentage of patients with ambulatory capacity [28]. Madigan and Wallace observed the same trend, but did not report on statistical significance [5]. Hägglund et al. (QAT-CS 14) found that a GMFCS grade III or higher was a significant risk factor for the development of moderate or severe scoliosis [3].

Yoshida et al. [19] (QAT-CS 18) could not demonstrate a significant association between the GMFCS grade and curve progression after the age of 20 years.

### 3.6. Factors Not Significantly Associated with Scoliotic Curve Progression after Growth Maturity

#### 3.6.1. Hip Instability

Four out of five studies [18,21,22,26] investigating the effect of hip instability on the progression of scoliosis conclusively showed that no correlation existed. All of these reports had a QAT-CS score above average.

Lee et al. [18] measured the migration percentage of the hips (MP, quantified by the method of Reimers [31]) in 184 study participants and found that hip instability (migration percentage > 33%) did not affect curve progression. This article scored 19 points on the QAT-CS scale, but included only 42 patients that had Risser’s grade 4 or 5. Oda et al. (QAT-CS 17) found no significantly different incidence of hip dislocation (MP ≥ 100%) between the groups with mild, moderate and severe curve progression beyond the age of 18 years [21]. In a study population of 106 CP patients with spastic quadriplegia, Senaran et al. (QAT-CS 17) found that unilateral hip dislocation (MP > 60%) did not affect the progression of scoliosis. However, the majority of the study population consisted of skeletally immature patients. Patients with bilaterally dislocated hips were excluded from this study [22]. Gu et al. (QAT-CS 15) also showed that a history of unilateral or bilateral hip dislocation was not a significant risk factor for curve progression. Their study population consisted mainly of skeletally immature patients [26].

One study by Yoshida et al. (QAT-CS 18) found a significant association between hip dislocation (both unilateral and bilateral) and curve progression during growth [19]. However, this association was not proved for progression of the curvature after growth maturity.

Two studies described the incidence of hip dislocation in the CP population, but did not report on curve progression in particular. Kalen et al. [28] found a higher incidence in patients with a scoliotic curve > 45°. Madigan and Wallace [5] also observed a higher incidence of hip dislocation when comparing CP patients with scoliosis to those without. The statistical significance of their results could not be confirmed, and the authors concluded that hip dislocation rather reflected a degree of neurological impairment more than it had an influence on the spinal curve.

#### 3.6.2. Pelvic Obliquity

Two studies (both QAT-CS 17) showed that pelvic obliquity does not influence the progression of scoliosis. Oda et al. found no statistically significant difference for the occurrence of pelvic obliquity between the groups with mild, moderate and severe curve progression [21]. Senaran et al. concluded that pelvic obliquity did not have an influence on an annual increase in scoliosis. Pelvic obliquity was found to progress over time at a faster rate in patients with a unilateral hip dislocation, but pelvic obliquity did not affect scoliotic curve progression [22]. Kalen et al. found a higher incidence of pelvic obliquity in adult patients with greater scoliotic curves, but the study design did not allow the drawing of conclusions on the effect of pelvic obliquity on curve progression during adulthood [28].

#### 3.6.3. Physiologic Type of CP

Only three articles, with a QAT-CS ranging from 16 to 18, attempted to find a correlation between the physiologic type of CP and curve progression in particular [11,23,25]. Two studies had a similar set-up and found statistically ambiguous results in a total study population that jointly counted 120 individuals with spastic, dystonic and mixed CP [23,25]. Patients with spasticity appeared to have a significantly higher risk for scoliosis progression beyond 40 degrees CA in both studies (odds ratio 3.26). However, when this risk factor was considered independently by statistical linear regression analysis, statistical significance could not be confirmed. Yoshida et al. did not find a significant association either between the type of CP and curve progression after skeletal maturity [19].

Thometz and Simon could not include enough patients with various types of CP to search for potential associations with curve progression [24]. Nonetheless, they investigated whether the anatomical distribution of spasticity could influence the progression of scoliosis after skeletal maturity. No statistically significant difference was found between spastic quadriplegic, hemiplegic or diplegic patients. One study (Madigan and Wallace) investigated patients with spastic, dyskinetic, ataxic and mixed-type CP and found the highest incidence of scoliosis in the group with spastic quadriplegics (75%). Yet, they did not report on the incidence of curve progression in this group [5].

#### 3.6.4. Type of Scoliotic Curve

Four studies investigated the influence of curve type on progression [8,20,24]. Yoshida et al. (QAT-CS 18) [19], Majd et al. (QAT-CS 17) [20] and Thometz and Simon (QAT-CS 16) did not find a statistically significant difference in curve progression between thoracic, thoracolumbar and lumbar scoliotic curves. The latter two studies included strictly skeletally mature patients (Risser 5).

Saito et al. (QAT-CS 13) found a faster curve progression in patients with thoracolumbar curves. The statistical significance of their findings was not reported. Also, a high incidence of severe functional impairment was identified as a potential cause of rapid scoliosis progression in that group [8].

#### 3.6.5. Positioning and Gravity

All of the three studies that reported on the effect of gravity and positioning scored below average on the QAT-CS scale. Two of them (QAT-CS 13, 10) could not prove an association of positioning or gravity with curve progression [5,8], and one study found a correlation that was arguable as a matter of cause–consequence (QAT-CS 13) [27].

Saito et al. (QAT-CS 13) concluded that gravity does not play an important role in rapid curve progression, as the incidence of progression was the highest in the group of bedridden patients whose spines were not subject to axial gravitational forces. Madigan and Wallace (QAT-CS 10) also stated with similar arguments that the severity of CP plays a more important part in aggravating scoliosis than gravity.

A study of 246 patients with severe CP (GMFCS V) (QAT-CS 13) found a statistically significant correlation between a preferred asymmetrical lying posture in the first year of life and the pattern of subsequent spinal deformity [27]. They put forward the idea that an interplay of biomechanical forces and gravity could influence spinal deformity. Yet, it was unclear whether lying posture was a cause or rather a consequence of asymmetric spinal deformity. Furthermore, this statistically significant association was not extrapolated to a potential effect on curve progression.

#### 3.6.6. Weight and Length

Gu et al. (QAT-CS 15) were the only study to investigate the effect of weight and length on curve progression. After correction for age, weight did not appear to be a significant predictor of scoliosis progression, nor did length. A total of 67 adolescents were included in this study, and the other 43 participants were children. The stage of skeletal maturity was not determined [26].

#### 3.6.7. Hip Surgery

A history of hip surgery did not affect the rate of curve progression in a study population of 184 subjects, consisting of 42 patients with Risser’s sign 4–5 (QAT-CS 19) [18]. Nevertheless, two studies by Bertoncelli et al. (QAT-CS 17, 16), in a total population of 120 participants, found that patients with previous hip surgery were significantly more likely to have scoliotic curves that progressed beyond 40° CA [23,25]. The significance was found by Fisher exact test and statistical regression analysis. However, the follow-up in these two studies did not continue beyond the age of 18 years, and no information on skeletal maturity was provided. The mean duration of follow-up was 5.3 years. As both of these studies had a lower methodological quality than the study by Lee et al. [18], previous hip surgery was not classified as a risk factor.

#### 3.6.8. Sex

The two studies that scored the highest in the quality assessment found no association between gender and curve progression (QAT-CS 18,19) [18,19]. These findings were contested by two other studies (QAT-CS 17, 16) by Bertoncelli et al. [23,25] that suggested an association between female sex and a higher risk of curve progression beyond 40 degrees. However, a *p*-value of 0.07 was found after a multicenter statistical analysis of this risk factor in a joint population of 120 participants.

#### 3.6.9. Epilepsy

Two of the included articles (QAT-CS 17, 16) were the first to identify intractable epilepsy in patients with CP as a potential risk factor for the development of severe scoliosis, but could not show a statistical significance [23,25]. When compared to CP patients without epilepsy, the group of patients with intractable epilepsy had a 2.7-fold risk of having scoliotic curves progressing beyond 40°. However, a calculation of statistical significance by logistic regression analysis yielded a *p*-value of 0.07 when the two study populations were evaluated as a whole. This implies that no strict statistical significance was found to identify epilepsy as an independent risk factor.

## 4. Discussion

The results of this review are relevant, as they provide a synthesis of all the available data on scoliotic curve progression after skeletal maturity in patients with cerebral palsy. A systematic strategy for the identification and interpretation of articles was combined with a strict assessment of the methodological quality of the retrieved studies. However, some limitations need to be acknowledged.

First, despite the high incidence of scoliosis in CP patients and the frequent occurrence of curve progression beyond skeletal maturity, only a limited quantity of data was available on this highly relevant clinical problem. After a very elaborate literature search and the application of eligibility criteria, only 15 original research articles on this topic were identified. None of the articles reached a level of evidence higher than III. As a result of the heterogeneity within the quite limited study populations of most articles (ranging from 34 to 272 participants), only small groups of patients with similar characteristics could be evaluated for comparison of curve progression.

Second, measurement methods of scoliosis differed between the included studies. Most articles measured the Cobb angle on anteroposterior radiographs of the study participants, which is a reliable method to quantify scoliosis [32]. However, the patient positioning to take these radiographs differed within and between study populations, depending on the physical capability of the patients to sit or stand. In only three studies, radiographs were systematically taken in the supine position. In the other studies, radiographs of some patients that required assistance to sit were taken in a sitting position. It has been suggested that a more accurate evaluation can be made with supine-position radiographs, as postural curves precipitated by sitting or standing are then eliminated [5,8,20,21].

Third, several included articles did not clearly determine the stage of skeletal maturity of their patients. Some articles estimated skeletal maturity by age; other articles described risk factors in a mixed population of both skeletally mature and immature patients. The articles with the higher QAT-CS scores evaluated the Risser sign on spine radiographs. Gupta et al. found that separate pelvis anteroposterior radiographs should be obtained to accurately visualize the radiographic anatomy needed to determine skeletal maturity [33]. Other authors suggest that a radiographic examination of the hand or elbow are more reliable than the Risser sign [34,35].

Fourth, in all the reviewed studies, patients who had received surgical treatment for scoliosis were excluded, or follow-up was terminated once patients received a treatment intervention. This is a type of selection bias that should be considered in the calculation of average annual scoliosis progression.

Fifth, the longitudinal data of the retrospective studies in this review were generally unbalanced, because an equal number of measurements was not available for all patients. Only one study incorporated between-subject variation by statistical means with a linear mixed model [18].

Sixth, during the review process, two authors were involved; however, they did not screen articles or collect data independently.

Given the limitations of this review, a prospective cohort would be the most advisable study design for future research. Prospective studies are needed to consolidate the current evidence for the two identified risk factors for curve progression after skeletal maturity (magnitude of the curve in adolescence and severity of motor impairment), and to reduce the risk of a type 2 error for the factors classified in this review as “no significant association found”.

The spastic quadriplegic subtype of CP correlates with a higher incidence of scoliosis [5,24], but no significant association with the progression of scoliosis has been proved [23,25]. An investigation of the association of spasticity with the progression of scoliosis in a group of individuals with the same GMFCS level could elucidate whether type of CP is an independent risk factor for curve progression.

More research is needed to clarify whether this is also applicable to spasticity and the progression of scoliosis. Bertoncelli et al. indeed found a higher risk for curve progression in the spastic subtype, but no significant association was found for spasticity as an independent risk factor [23,25].

Intrathecal baclofen (ITB) has been proved effective in reducing spasticity and is a common treatment for adolescents or adults with CP [36]. In this respect, it is noteworthy that the effect of intrathecal baclofen treatment on scoliosis progression remains unclear [37,38]. Ginsberg et al. observed accelerated curve progression after ITB pump placement [39]. Whether this is due to ITB, accelerated adolescent growth or the finding that ITB pumps are placed in patients severely affected by spasticity is still open to debate [36].

A systematic review by Loeters et al. tried to translate the statistical association between functional motor deficit and the progression of scoliosis into mechanisms causing progression. They stated that the combination of a limited range of motion and poor postural control contributes to the progression of scoliosis in patients with severe CP [9]. Porter et al. stated that there is no clear evidence that scoliosis is primarily driven by muscle imbalance [27].

Asymmetrical bone growth occurs under asymmetrical load. In that respect, insufficient trunk control can contribute to the progression of scoliosis. Both arguments are used by Rutz et al. to stress the importance of early bracing treatment in CP patients with scoliosis [40]. Concordantly, the tonification of the back musculature through ambulation and an upright posture protect against the development of severe scoliosis during growth [25]. As growth plays an important role in the development of scoliosis through truncal imbalance, risk factors for curve progression in children and adolescents may differ from risk factors in skeletally mature patients. This stresses the importance of determining the stage of skeletal maturity in future studies.

Given the high prevalence of epilepsy among children with cerebral palsy (up to 40%) [41], a further investigation of this potential risk factor is needed. Bertoncelli et al. [23,25] were the first and only authors to identify epilepsy as a potential risk factor for the development of severe scoliosis in CP patients. An odds ratio of 2.7 was calculated; however, it was not statistically significant.

Oda et al. [21] indicated that a CA of 50° at skeletal maturity might not be a suitable benchmark to differentiate between fast and slow progressors, as the mean CA in their study group with a moderate curve progression reached approximately 50° at completion of growth.

This review shows that there is no association between hip instability and the progression of scoliosis. Furthermore, muscle release below the iliac crest does not halt the progression of scoliosis [42]. This agrees with the hypothesis that scoliosis and pelvic obliquity are rather correlated with the severity of neurologic involvement than with the mechanics of hip dislocation [43]. Yet, Porter et al. observed a higher occurrence of hip subluxation on the same side as the convexity of the spinal deformity [44].

Yoshida et al. found a significant association between hip dislocation and the magnitude of the scoliotic curve during growth; however, the association with curve progression after skeletal maturity was not investigated. [12,19]. Lee et al. specifically investigated the annual curve progression and did not find an association with hip dislocation. Future studies with a follow-up after skeletal maturity are required to elucidate whether the influence of hip dislocation on the scoliotic curve during growth also persists in adulthood [18].

### 4.1. Strategy for Clinical Follow-Up

The magnitude of the scoliotic curve and a high GMFCS level appeared significant risk factors in this review. The included articles found a mean annual curve progression ranging from 0.5 degrees to 0.8 degrees per year for patients with a scoliotic curve < 50° CA at the end of adolescence. In the case of severe motor impairment or a curve exceeding 50 degrees, the annual increase ranged from 1.4 to 3.5 degrees per year. Taking into account that the gold standard for a true change in CA is a measured change of 5 degrees [45], a radiographic follow-up once every 3 years is recommended for skeletally mature patients with CP that have a scoliotic curve of >50° at the end of growth or with a GMFCS level IV-V. For patients with none of these risk factors, a regular follow-up once every 5 years after skeletal maturity will be sufficient to monitor curve progression. It is important to remark that the mathematical progression of scoliosis most likely does not occur in a linear fashion, but rather as a quadratic function, with a larger annual increment when the absolute Cobb angle increases [26].

### 4.2. Timing of Surgery

An increasing scoliotic curve leads to functional deterioration in CP patients [20]. Conservative treatment by means of bracing can improve functioning, but does not prevent curve progression [46,47,48]. Cloake et al. stated that surgery should be considered for patients with large curves (>50°), a continuing curve progression beyond skeletal maturity and patients with significant curves resulting in severe functional impairment [47]. A recent study from Miyanji et al. showed that scoliosis surgery improves the health-related quality of life in CP patients as estimated by caregivers until 5 years post-operatively. Despite a relatively high complication rate, the benefits of surgery seem to outweigh the risks for severely impaired CP patients [49]. The evaluation of risk factors, such as a large curve at the end of adolescence or a severe motor deficit (GMFCS IV-V), can help to estimate future curve progression and identify patients that would benefit from early surgical intervention. A regular radiographic follow-up at the recommended 3- or 5-year intervals can aid in differentiating between fast progressors and slow progressors, thus facilitating decision making on the necessity of surgical treatment on an individual basis.

## 5. Conclusions

The progression of a scoliotic curve after skeletal maturity is a common finding in patients with cerebral palsy. CP patients with a Cobb angle greater than 40–50 degrees at the end of adolescence or with a severe motor deficit (an inability to walk or GMFCS IV-V) have a significantly higher risk of curve progression in adulthood. If at least one of both risk factors is present, the average annual increase is estimated at 1.4–3.5 degrees per year. In patients without these risk factors, scoliotic curves are unlikely to progress significantly beyond skeletal maturity. The estimates of annual increase for those patients range from 0.1 to 0.8 degrees per year. This review shows that scoliosis in cerebral palsy is not a uniform entity. It behaves very diversely, depending on the severity of CP. On the same grounds, the natural history of scoliosis in patients with CP differs from patients with other neuromuscular disorders, such as spinal muscular atrophy or Duchenne muscular dystrophy. The umbrella term neuromuscular scoliosis to designate non-idiopathic scoliosis should therefore be used with great caution, as it only refers to a morphological finding in patients with a certain comorbidity and does not relate to a type of spinal deformity with a uniform pathophysiology, characteristics and functional or therapeutic consequences.

## Figures and Tables

**Table 1:** Database search algorithms.

PubMed—Searched on 1 May 2024	
1	Cerebral palsy [MeSH Terms] or Cerebral palsy/complications [MeSH Terms]
2	“cerebral palsy” or (“cerebral” and “palsy”) or “spastic diplegi *” or “spastic hemiplegi *” or “spastic paraplegi *” or “spastic quadriplegi *” or “spastic tetraplegi *” or (“athetoid” and “palsy”) or “dyskinetic” or (“ataxi *” and “palsy”) or (“mixed” and “palsy”)
3	1 or 2
4	Scoliosis [MeSH Terms] or Scoliosis/complications [MeSH Terms]
5	“scoliosis” or “spinal deformit *” or “neuromuscular scoliosis”
6	4 or 5
7	Natural history [MeSH Terms] or Disease progression [MeSH Terms]
8	“natural history” or (“natural” and “history”) or “disease progression” or (“disease” and “progression”) or “progression” or “untreated”
9	7 or 8
10	Risk factors [MeSH Terms] and Disease progression [MeSH Terms]
11	“risk factor *” or (“risk” and “factors”) and (“disease progression” or “progression” or (“disease” and “progression”))
12	10 or 11
13	Risk factors [MeSH Terms]
14	“risk factors” or (“risk” and “factors”)
15	3 and 6 and (9 or 12 or 14)
EMBase—searched on 1 May 2024	
(‘cerebral palsy’/exp OR ‘cerebral palsy’) AND (‘scoliosis’/exp OR ‘scoliosis’) AND (‘natural history’/exp OR ‘natural history’ OR ‘disease progression’/exp OR ‘disease progression’)	
Cochrane Library—searched on 1 May 2024	
cerebral palsy AND scoliosis AND (natural history OR disease progression)	

* Asterisk = truncation method used in PubMed allowing any group of characters (or no character) to be added to the search term to broaden the search.

**Table 2:** Assessment of methodological quality in accordance with the Quality Assessment Tool for Case Series by IHE [17].

		**STUDY OBJECTIVE**	
1		Is the objective of the study stated clearly?	0 = no 1 = yes
		**STUDY POPULATION**	
2		Are the characteristics of the participants included in the study?	
	2a	Age	0 = no information provided 1 = information restricted to age range of participants 2 = detailed information on age of participants
	2b	Type of scoliotic curve	0 = no information on curve pattern 1 = information on curve type or localization of curve
	2c	Functional status	0 = no information on functional status of participants 1 = information on walking ability or GMFCS-class
	2d	Physiologic type of cerebral palsy	0 = not mentioned 1 = information on distribution of physiologic types among participants
	2e	Pelvic obliquity	0 = no information provided on presence of pelvic obliquity 1 = information on presence of pelvic obliquity
	2f	Hip dislocation	0 = no information on hip dislocation 1 = information on presence of hip dislocation
	2g	Skeletal maturity	0 = no clear definition of skeletal maturity used 1 = determined by age, not by radiographic findings 2 = determined by Risser’s classification
	2h	Other comorbidities (e.g. epilepsy, mental retardation)	0 = no information on the presence of other comorbidities of the studied groups 1 = presence or absence of 1 or more comorbidities mentioned
3		Were the cases collected in more than one centre?	0 = no 1 = yes
4		Are the eligibility criteria (inclusion and exclusion criteria) for entry into the study explicit and appropriate?	
	4a	Scoliosis	0 = incomplete information on inclusion and exclusion criteria 1 = adequate description of exclusion criteria, inclusion by review of available radiographs or clinical assessment 2 = adequate description of exclusion criteria, inclusion by systematic radiographic screening of participants
	4b	Were participants recruited consecutively?	0 = no 1 = yes 2 = consecutive recruitment of participants in a population-based sample
	4c	Did participants of the studied groups enter the study at a similar point in the disease?	0 = participants entered the study at different stages of skeletal maturity 1 = all the participants entered the study at the same stage of skeletal maturity
		**OUTCOME MEASURE**	
5		Are the outcome measures clearly defined in the introduction or methods section?	0 = no description of outcome measures 1 = adequate description of outcome measures
6		Were relevant outcomes appropriately measured with objective methods?	0 = no description of measurement method or no radiographic measurement of scoliotic curve 1 = radiographic measurement of scoliotic curve by method of Cobb, not all of the measurements in supine position 2 = radiographic measurement of scoliotic curve by method of Cobb, all measurements in supine position
		**STATISTICAL ANALYSIS**	
7		Were the statistical tests used to assess the relevant outcomes appropriate?	0 = no information in methods section on used statistical tests 1 = information on used statistical tests
		**RESULTS AND CONCLUSIONS**	
8		Was the length of follow-up reported?	0 = no information on length of follow-up or study without follow-up of participants 1 = mean duration of follow-up mentioned
9		Was the loss to follow-up reported?	0 = no information on loss to follow-up or study without follow-up of participants 1 = information provided on loss to follow-up
10		Does the study provide estimates of the random variability in the data analysis of relevant outcomes?	0 = no information on random variability 1 = information on statistical significance of data
11		Are the conclusions of the study supported by results?	0 = one or more conclusions were not drawn by logical interpretation of results 1 = all of the conclusions were drawn by logical interpretation of results
12		Are both competing interests and sources of support for the study reported?	0 = no disclosure of conflicts of interests 1 = disclosure of conflicts of interests
		TOTAL SCORE	max. 26 points

**Table 3:** Overview of the methodological quality of the included studies in accordance with the modified Quality Assessment Tool for Case Series (QAT-CS). The studies are listed in the order of their QAT-CS score.

			Lee et al. (2016) [18]	Yoshida et al. (2018) [19]	Majd et al. (1997) [20]	Oda et al. (2017) [21]	Senaran et al. (2006) [22]	Bertoncelli et al. (2018) [23]	Thometz and Simon (1988) [24]	Bertoncelli et al. (2017) [25]	Gu et al. (2011) [26]	Hägglund et al. (2018) [3]	Saito et al. (1998) [8]	Porter et al. (2008) [27]	Kalen et al. (1992) [28]	Madigan and Wallace (1981) [5]	Robson (1968) [29]
1		Study objective	1	1	1	1	1	1	1	1	1	1	1	1	0	1	0
2		Characteristics of the participants															
	2a	Age	1	2	1	2	1	1	1	1	1	1	1	1	1	1	2
	2b	Type of scoliotic curve	1	1	1	0	1	0	1	0	1	0	1	0	0	1	1
	2c	Functional status	1	1	1	1	1	1	1	1	1	1	1	1	1	1	0
	2d	Physiologic type of cerebral palsy	1	1	1	0	1	1	1	1	1	0	1	0	1	1	1
	2e	Pelvic obliquity	1	0	1	1	1	0	0	0	0	0	0	1	1	0	0
	2f	Hip dislocation	1	1	0	1	1	0	0	0	1	0	0	1	1	1	0
	2g	Skeletal maturity	2	1	2	1	0	0	2	0	0	0	0	0	0	0	0
	2h	Other comorbidities	0	0	1	0	0	1	0	1	0	0	1	0	1	0	0
3		Collection of cases	0	0	0	0	0	1	0	0	0	1	0	1	0	0	1
4		Eligibility criteria															
	4a	Scoliosis	1	2	1	1	1	1	1	1	1	1	2	1	0	2	1
	4b	Recruitment of participants	1	0	0	1	1	2	0	2	1	2	0	1	0	0	0
	4c	Stage of skeletal maturity at initial follow-up	0	0	1	0	0	0	1	0	0	0	0	0	1	0	0
5		Outcome measures	1	1	1	1	1	1	1	1	1	1	1	1	1	1	0
6		Measurement method	1	2	1	2	1	1	2	1	1	1	2	0	1	1	0
7		Statistical analysis	1	1	1	1	1	1	0	1	1	1	0	1	0	0	1
8		Length of follow-up	1	1	1	1	1	1	1	1	1	0	1	0	0	0	0
9		Loss to follow-up reported	1	0	0	0	1	1	1	1	0	1	1	0	0	0	0
10		Estimates of random variability	1	1	1	1	1	1	1	1	1	1	0	1	1	0	1
11		Conclusions	1	1	1	1	1	1	1	1	1	1	0	1	1	0	0
12		Competing interests	1	1	0	1	1	1	0	1	1	1	0	1	0	0	0
		**TOTAL SCORE** **(max. 26 points)**	19	18	17	17	17	17	16	16	15	14	13	13	11	10	8
		mean = 16															
		median = 16															

**Table 4:** Overview of the characteristics of the included articles and their associations with curve progression after skeletal maturity. The studies are listed in the order of their QAT-CS score.

	Study Design	QAT-CS	*N*		Age	Type of CP	Severity of CP	Definition of Skeletal Maturity	Progression Rate or Incidence	Curve Magnitude	Severity of Motor Deficit	Hip Instability	PO	Hip Surgery	Type of CP	Type of Curve	Positioning and Gravity	Weight and Length	Sex	Epilepsy
Lee et al. (2016) [18]	retrospective, level IV	19	184	consecutive clinical sample	2.7–29.3 y	spastic CP	GMFCS I-V	Risser 0–5	3.4°/y if GMFCS IV-V		+, GMFCS IV-V 3.4°/y GMFCS I-III 0°/y	-		-					-	
Yoshida et al. (2018) [19]	retrospective, Level IV	18	113	single centre	0–32 y	mixed	GMFCS I-V	age >18 y	32.5% progression after 20 y of age	-, n.s.	-, *p* = 0.33 in multivariate analysis	+, during growth, not in adulthood			-	-			-	
Majd et al. (1997) [20]	prospective, level IV	17	56	institutionalised	15–53 y	mixed, 68% spastic	mixed	Risser 5	2.5–3.5°/y							-				
Oda et al. (2017) [21]	retrospective, level IV	17	34	consecutive clinical sample	initial 10–18 y, final 18–30 y	ND	GMFCS IV-V	age	74%	+, 3.0°/y if >50°; 0.1–0.5°/y if <50°		-, for MP > 100%	-							
Senaran et al. (2006) [22]	retrospective, level IV	17	106	consecutive clinical sample	3–18 y	spastic quadriplegic CP	mixed	ND				-, for MP > 60%	-							
Bertoncelli et al. (2018) [23]	cross-sectional, descriptive, level IV	17	120	multicentre clinical sample	12–18 y	mixed	GMFCS II-V	ND			- OR 3.19 but no independent RF			+	- OR 3.26 but no independent RF				-, *p* = 0.07	-, *p* = 0.07
Thometz and Simon (1988) [24]	retrospective, level IV	16	51	institutionalized	mean age at SM = 23.7 y, duration of FU after SM = mean 16.3 y	mixed	mixed	Risser 5	78% 1–3°/y	+, 0.8°/y if <50 1.4°/y if >50	+ walking ability									
Bertoncelli et al. (2017) [25]	cross-sectional, descriptive, level IV	16	70	clinical sample	12–18 y	mixed	GMFCS II-V	ND			no independent RF			+	no independent RF			+, *p* = 0.02		+, *p* = 0.02
Gu et al. (2011) [26]	retrospective, level IV	15	110	institutionalized	0.5–18.1 y	spastic quadriplegic CP	GMFCS V	ND		+, if >40° at 12 y		- unilateral or bilateral hip dislocation						-		
Hägglund et al. (2018) [3]	prospective, level III	14	962	multicentre	0–25 y	mixed	GMFCS I-V	ND	incidence of scoliosis continues to increase between 15–25 y of age		+ GMFCS III and above, *p* < 0.04								-, *p* = 0.10	
Saito et al. (1998) [8]	retrospective, level IV	13	37	institutionalized	initial 1–15 y, final 15–36 y	spastic CP	mixed	age	73%	+, if >40 at 15 y	+									
Porter et al. (2008) [27]	retrospective, level IV	13	246	multicentre clinical sample	1–19 y	ND	GMFCS V	ND									-			
Kalen et al. (1992) [28]	cross-sectional, level IV	11	56	institutionalized	29–67 y	spastic CP	mixed	ND			+, walking ability with greater scoliotic curve (not progression), s.n.r.	+, n.s.	+, with greater scoliotic curve (not progression) s.n.r.							
Madigan and Wallace (1981) [5]	cross-sectional, descriptive level IV	10	272	institutionalized	1–68 y	mixed	mixed	ND			+, n.s.	+, n.s.								
Robson (1968) [29]	cross-sectional, descriptive, level IV	8	152	multicentre, institutionalized	11–47 y	mixed	ND	ND	incidence of scoliosis increases with age											

ND, not defined; RF, risk factor; SM, skeletal maturity; FU, follow-up; PO, pelvic obliquity; n.s., not significant; s.n.r., significance not reported. + = assocation with curve progression. - = no significant assocation found.

## Data Availability

No new data were created or analyzed in this study. Data sharing is not applicable to this article.
